# An Enhanced “Trapping−Conversion” Function Enables Ultrastable Potassium Ion Storage

**DOI:** 10.1002/advs.202503332

**Published:** 2025-05-08

**Authors:** Zhongquan Wang, Bangjun Wu, Zhenping Qiu, Qingguang Zeng, Aruuhan Bayaguud, Huirong Wang, Zheng Liu, Yiju Li, Yelong Zhang

**Affiliations:** ^1^ School of Renewable Energy Inner Mongolia University of Technology Ordos 017010 China; ^2^ Inner Mongolia Key Laboratory of New Energy and Energy Storage Technology Hohhot 010051 China; ^3^ School of Materials Science and Engineering Inner Mongolia University of Technology Hohhot 010051 China; ^4^ School of Applied Physics and Materials Wuyi University Jiangmen Guangdong 529020 China; ^5^ Institute of Carbon Peaking and Carbon Neutralization Wuyi University Jiangmen Guangdong 529020 China; ^6^ Department of Mechanical and Energy Engineering Southern University of Science and Technology Shenzhen 518055 China

**Keywords:** MXene, PbTe, P‐doping, potassium‐ion batteries, shuttle effect

## Abstract

Metal chalcogenides (MCs) have emerged as promising candidates for potassium ion battery (KIB) anode materials, yet the sluggish redox kinetics and notorious shuttle effect inescapability lead to inferior rate performance and poor cyclability. Herein, a P‐doped PbTe/MXene (P‐PbTe/MXene) superstructure is rationally constructed by decorating PbTe on MXene via a hydrothermal reaction and followed by bifunctional P‐doping, where P heteroatoms enter both PbTe and MXene lattice. The P‐PbTe/MXene anode shows enhanced reaction kinetics and suppressed shuttle effect of polytellurides due to the enhanced chemical adsorption stemming from the low energy gaps between the d‐band center and the p‐band center of P‐MXene. As a result, the P‐PbTe/MXene superstructure shows superior potassium storage properties, including high reversible capacity (289.1 mAh g^−1^ at 0.2 A g^−1^ after 200 cycles), outstanding rate performance (151.3 mAh g^−1^ at 20 A g^−1^), and ultrastable cyclability (180.1 mA h g^−1^ at 2.0 A g^−1^ after 2000 cycles) in half battery. Also, the P‐PbTe/MXene anode exhibits high energy density (186.0 Wh kg^−1^ at 0.1 A g^−1^) and excellent bending stability in soft‐package full cells.

## Graphical abstract

An enhanced “trapping−conversion” function is proposed to improve redox kinetics and suppress shuttle effect of polytellurides for KIBs anode. Due to low energy gaps between the d‐band center and the p‐band center of P‐MXene, the engineered P‐PbTe/MXene anode demonstrates high reversible capacity, excellent rate capability, and superb cyclability, as well as reliable operation in flexible potassium ion full cell.

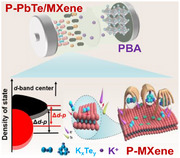

## 1 Introduction

Developing more efficient energy storage devices is one critical initiative to realize the goal of carbon neutrality.^[^ ^1^ ^]^ Among the emerging battery technologies, potassium ion batteries (KIBs) are considered promising alternatives to LIBs due to abundant potassium sources, cost competitiveness, and similar electrochemical mechanisms.^[^ ^2^ ^]^ Unfortunately, the sluggish reaction kinetics and huge volume variation of anode materials induced by the large ionic radius of K^+^ (1.38 Å) result in poor rate capability and cycling performance.^[^ ^3^ ^]^ It is therefore essential to develop ideal KIB anode materials with excellent cycle and rate performance.

A large number of carbonaceous materials,^[^ ^4^ ^]^ metal chalcogenides (MCs),^[^ ^5^ ^]^ metal oxides,^[^ ^6^ ^]^ and organic materials^[^ ^7^ ^]^ have been intensively investigated as anode materials for KIBs. Among them, MCs are attracting extensive attention due to their high theoretical capacity and low cost.^[^ ^8^ ^]^ Nevertheless, similar to other conversion‐alloying electrodes, severe volume variation of MCs during battery operation generally results in electrode structure collapse and subsequent rapid capacity decay.^[^ ^9^ ^]^ Especially, the dissolution and shuttling of soluble polytellurides intermediates and their slow transformation reaction kinetics during cycling can cause accelerated capacity fade and unsatisfactory rate capability.^[^ ^10^ ^]^ To solve those issues, extensive studies have been dedicated to improving rate performance and cycle life through structure engineering,^[^ ^11^ ^]^ interface engineering,^[^ ^12^ ^]^ and doping engineering.^[^ ^13^ ^]^ It is well known that Pb‐based materials are generally used because of their earth‐abundance, cost‐effectiveness, and reliability for energy storage. PbTe, a narrow‐gap semiconductor in the Pb chalcogenide family, has weaker metal‐nonmetal bonding than PbS and PbSe because of the Te anion's higher atomic number.^[^ ^14^ ^]^ This facilitates the conversion reaction and makes PbTe particularly appealing in KIBs. Unfortunately, no research has been conducted so far to investigate this promising candidate anode for KIBs, and its potassium storage mechanism remains unveiled.

MXene has been widely exploited in electrochemical energy storage for its high conductivity, adjustable terminal group, and unique layered structure.^[^ ^15^ ^]^ In this work, PbTe is grown on the surface of MXene, followed by subsequent P doping, resulting in the formation of a novel superstructure (P‐PbTe/MXene). The P heteroatoms are doped into both PbTe and MXene (P‐MXene) of this superstructure, offering significant advantages as a promising anode material for KIBs: i) The hierarchical P‐PbTe/MXene with rich Te vacancy defects can offer multiple ion diffusion pathways and abundant active sites, which significantly promote the K^+^ storage kinetics; ii) The P‐MXene substrate exhibits high adsorption and catalytic capacity toward polytellurides intermediates, which can effectively suppress shuttling and accelerate transformation during repeated cycling. As a result, the P‐PbTe/MXene anode delivers a high reversible capacity of 289.1 mAh g^−1^ after 200 cycles at 0.2 A g^−1^, remarkable rate capability of 151.3 mAh g^−1^ at 20 A g^−1^, and a high capacity retention of 180.1 mAh g^−1^ after 2000 cycles at 2.0 A g^−1^. Theoretical calculations show that P‐PbTe/MXene has high electronic conductivity, strong adsorption and catalytic capacity, and low ion diffusion energy barriers. The potassiation of P‐PbTe/MXene undergoes reversible intercalation‐conversion‐alloying reaction pathways. Importantly, a soft package full cell with P‐PbTe/MXene anode and Prussian blue potassium (KPB) cathode delivers a high energy density of 186.0 Wh kg^−1^ at 0.1 A g^−1^, superb cycling stability of 107.9 mAh g^−1^ at 0.1 A g^−1^ after 400 cycles, and excellent flexibility.

## 2 Results and Discussion

**Figure 1:**
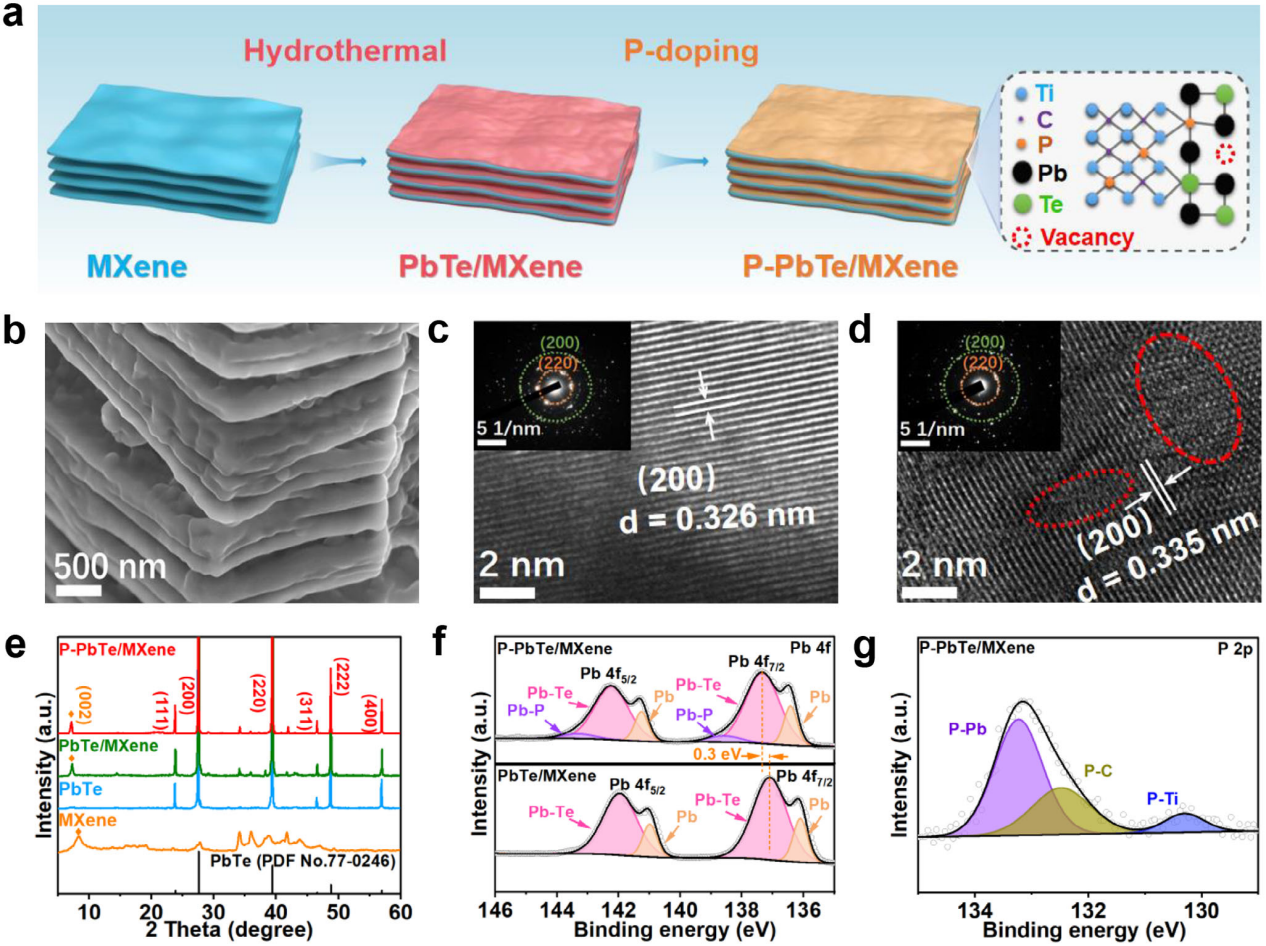
a) Synthetic scheme and b) SEM image of P‐PbTe/MXene. HRTEM images with inset of corresponding SAED patterns for c) PbTe/MXene and d) P‐PbTe/MXene, e) XRD patterns of P‐PbTe/MXene, PbTe/MXene, PbTe, and MXene. High‐resolution f) Pb 4f and g) P 2p XPS spectrum of P‐PbTe/MXene.

The synthesis process of the P‐PbTe/MXene superstructure is schematically illustrated in **Figure** **1**a. Initially, Ti_3_C_2_T_x_ MXene was prepared via selective chemical etching of Al layers from the Ti_3_AlC_2_ MAX phase in a hydrofluoric acid (HF) solution (Figure S1, Supporting Information). The PbTe/MXene was synthesized through a facile hydrothermal reaction of lead acetate ((CH_3_COO)_2 _Pb), hydroxylamine hydrochloride (NH_2_OH·HCl), sodium tellurite (Na_2_TeO_3_), sodium borohydride (NaBH_4_), and MXene at 200 °C. Finally, P‐PbTe/MXene was synthesized by annealing PbTe/MXene with sodium hypophosphite (NaH_2_PO_2_) as a P source at 450 °C under an Ar atmosphere.

After the hydrothermal reaction, the PbTe nanoparticles are densely and uniformly coated on MXene surface, forming the mille crepe‐like PbTe/MXene (Figure S2, Supporting Information). This hierarchical structure is still well retained in P‐PbTe/MXene after P doping (Figure 1b; Figure S3a, Supporting Information). Moreover, the elemental mapping (Figure S3b, Supporting Information) shows the uniform distribution of Pb, Te, Ti, C, and P elements in the P‐PbTe/MXene, further confirming the P‐doping is homogenous throughout the entire superstructure. The mass contents of P element and PbTe in P‐PbTe/MXene are determined to be 3.2% and 42.0% based on P/Ti and Pb/Ti ratios, respectively (Figure S3c, Supporting Information).

The high‐resolution transmission electron microscope (HRTEM) images and the corresponding selected area electron diffraction (SAED) patterns reveal the presence of cubic‐phase PbTe in both PbTe/MXene and P‐PbTe/MXene, as evidenced by the lattice fringes of and diffraction rings of (200) and (220) planes (Figure 1c,d).^[^ ^16^ ^]^ Besides, abundant atomic vacancies and defects in P‐PbTe/MXene can be observed (dashed red circles).^[^ ^17^ ^]^ The P‐PbTe phase is verified by the X‐ray diffraction (XRD) technique (Figure 1e), and the peaks located at 23.8°, 27.5°, 39.3°, 46.5°, 48.7°, and 56.9° can be indexed to the (111), (200), (220), (311), (222), and (400) planes of PbTe (PDF No.77‐0246), respectively.^[^ ^18^ ^]^ Besides, the (002) peak of MXene is shifted toward lower angles after compositing and P doping, suggesting an enlarged MXene layer spacing.^[^ ^19^ ^]^

X‐ray photoelectron spectroscopy (XPS) measurements are utilized to examine the chemical environments of the samples. The survey spectra identify the co‐existence of Ti, C, Pb, Te, and P elements in P‐PbTe/MXene (Figure S4a, Supporting Information). For the high‐resolution Pb 4f spectrum of P‐PbTe/MXene (Figure 1f), the two strong peaks at 137.3 and 142.2 eV can be attributed to the Pb‐Te bond.^[^ ^20^ ^]^ Two weak satellite peaks at 136.4 and 141.2 eV can be observed, which are likely caused by a trace amount of Pb.^[^ ^21^ ^]^ Moreover, two peaks at 138.6 and 143.4 eV are related to the Pb‐P bond,^[^ ^22^ ^]^ further demonstrating the successful P‐doping. As shown in the high‐resolution Te 3d XPS spectrum of P‐PbTe/MXene (Figure S4b, Supporting Information), the fitting peaks located at 575.1/585.6, 571.9/582.4, and 572.9/583.4 eV are assigned to the Te‐O, Te‐Pb, and Te‐Ti bonds, respectively.^[^ ^23^ ^]^ The existence of the Te‐O peaks may be caused by the exposure of the sample under ambient conditions.^[^ ^24^ ^]^ In the high‐resolution P 2p spectrum of P‐PbTe/MXene (Figure 1g), the peaks of P‐Pb (133.2 eV), P‐C (132.4),^[^ ^25^ ^]^ and P‐Ti (130.3)^[^ ^26^ ^]^ are observed, demonstrating that P dopant atoms are incorporated not only into PbTe but also into MXene. Compared with PbTe/MXene, the Pb 4f, both peaks of Pb 4f and Te 3d in P‐PbTe/MXene slightly shift toward higher binding energy, implying a change of electronic structure and redistribution of spatial charges due to the P doping.^[^ ^27^ ^]^

The presence of Te vacancies is further confirmed by electron paramagnetic resonance (EPR) spectroscopy analysis. Compared with PbTe and PbTe/MXene, a stronger EPR signal of Te vacancy at *g* = 2.003 has been observed in P‐PbTe/MXene, indicating the enriched vacancy content (Figure S5a, Supporting Information).^[^ ^28^ ^]^ The Brunauer‐Emmett‐Teller (BET) analysis (Figure S5b, Supporting Information) reveals a significant enhancement in the specific surface area of P‐PbTe/MXene (142.1 m^2^ g^−1^) compared to control samples, including PbTe/MXene (98.7 m^2^ g^−1^), pure PbTe (52.5 m^2^ g^−1^), and pristine MXene (45.4 m^2^ g^−1^). The higher surface area for P‐PbTe/MXene is attributed to the P doping treatment and the enlarged interlayer distance of MXene.^[15d]^

**Figure 2:**
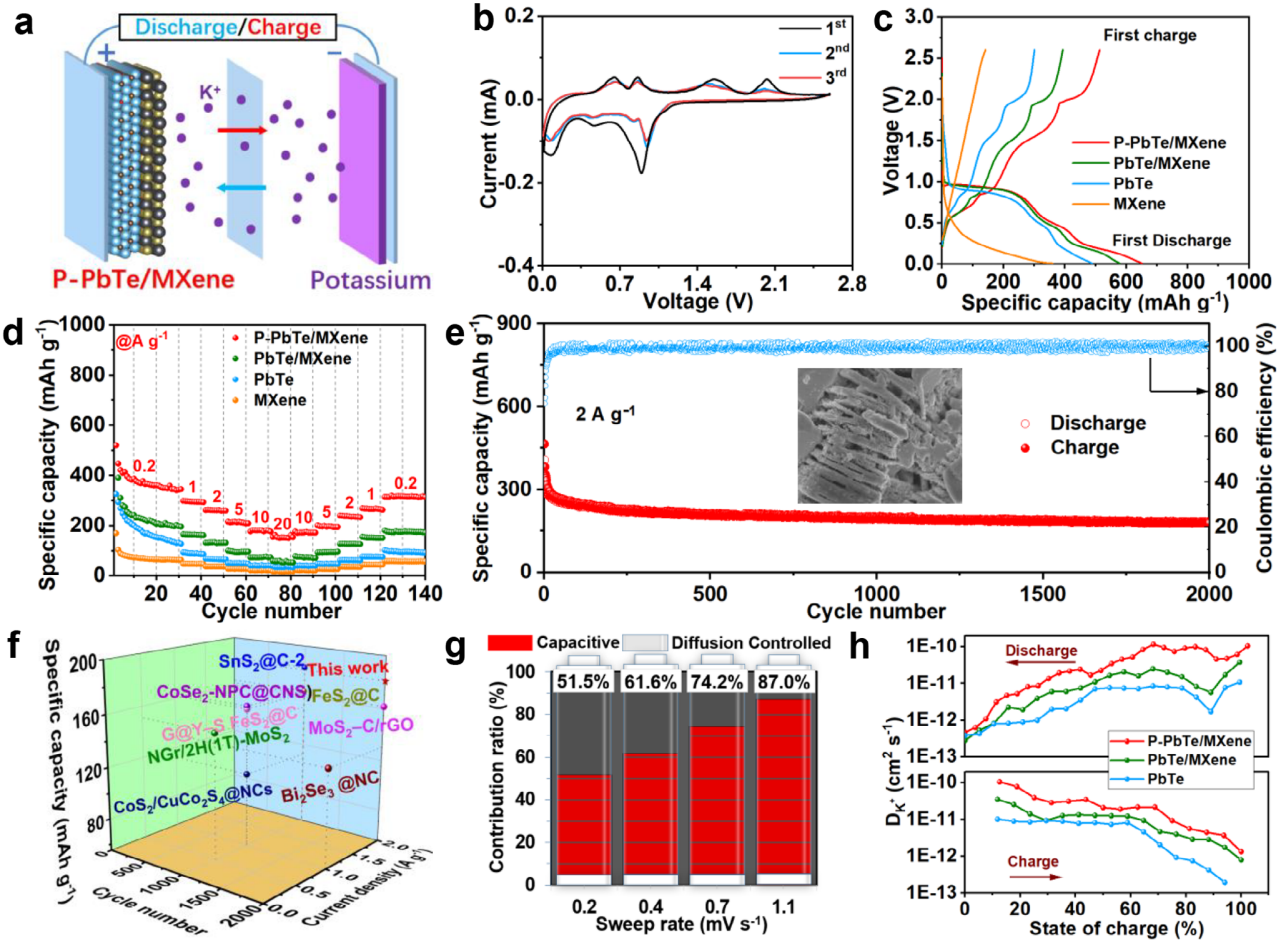
a) Schematic of P‐PbTe/MXene‐based half‐battery, b) CV curves at a scan rate of 0.1 mV s^−1^ for the initial three cycles, c) initial GCD curves at 0.2 A g^−1^, d) cycling performance at 2 A g^−1^, and e) rate capability of P‐PbTe/MXene. f) Comparison of selected materials for long‐term cycling performance. g) Normalized contribution ratio of capacitive capacity at differing scan rates and h) the calculated diffusion coefficient of K^+^ ion during the potassiation/depotassiation processes for P‐PbTe/MXene.

The prepared P‐PbTe/MXene superstructure was assembled into a CR2032‐type battery with a K metal foil as a counter electrode (**Figure** **2**a). The large reduction peak at ≈0.9 V in the initial cathodic scan corresponds to the solid electrolyte interface (SEI) film growth, the K^+^ ion intercalation, and the conversion of PbTe into Pb and K_2_Te (Figure 2b).^[^ ^21^, ^29^ ^]^ The two reduction peaks at 0.5 and 0.1 V are related to the alloying reactions for the formation of K_4_Pb_9_ and KPb.^[^ ^30^ ^]^ During the following anodic scanning, the two oxidation peaks at 0.6 and 0.9 V are associated with the dealloying process of the K‐Pb alloy. The two oxidation peaks observed at 1.5 and 2.0 V are attributed to the multistep transformation from K_2_Te and Pb to PbTe. The cyclic voltammetry (CV) profiles curves well overlap between the 2^nd^ and 3^rd^ cycles, implying good reversibility and structure stability of the P‐PbTe/MXene electrode.^[^ ^31^ ^]^

The galvanostatic charge‐discharge profiles of the P‐PbTe/MXene electrode for the first cycle at 0.2 A g^−1^ are shown in Figure 2c. The initial discharge/charge specific capacity of the P‐PbTe/MXene is 651.0/513.4 mAh g^−1^ with a Coulombic efficiency (CE) of 78.9%, much larger than those of PbTe/MXene (578.4/393.0 mAh g^−1^, 67.9%), PbTe (488.5/301.0 mAh g^−1^, 61.6%), and MXene (360.0/141.5 mAh g^−1^, 39.3%). The primary capacity fade mechanism originates from irreversible electrochemical reactions at the electrode‐electrolyte interface, predominantly attributed to SEI film formation during initial cycling.^[^ ^32^ ^]^ After 200 cycles, the P‐PbTe/MXene keeps a reversible capacity of 289.1 mAh g^−1^ (Figure S6, Supporting Information), much larger than those of PbTe/MXene (147.0 mAh g^−1^), PbTe (30.2 mAh g^−1^), and MXene (99.4 mAh g^−1^). The rate performance was evaluated at various current densities ranging from 0.2 to 20 A g^−1^ (Figure 2d). Compared with the PbTe/MXene, PbTe, and MXene anodes, the P‐PbTe/MXene can release much higher reversible capacities of 345.6, 292.8, 259.3, 208.4, 176.1, and 151.3 mAh g^−1^ at 0.2, 1, 2, 5, 10, and 20 A g^−1^, respectively. Besides, the reversible capacity of P‐PbTe/MXene can be restored to 316.4 mAh g^−1^ upon current density reset to 0.2 A g^−1^, indicating excellent reversibility and rapid reaction kinetics. Figure 2e presents long‐term cycling stability. The preserved capacity is 180.1 mAh g^−1^ after 2000 cycles with a capacity fading rate of only 0.0408% per cycle at a high current density of 2 A g^−1^, surpassing that of most other metal chalcogenides‐based materials for KIBs (Figure 2f; Table S1, Supporting Information).

The contribution of capacitance was analyzed by performing CV at different scan rates (Figure S7, Supporting Information), exhibiting one cathodic (peak 1) and two anodic peaks (peak 2 and peak 3). The peak current value (*i*) and scan rate (*v*) satisfy the following relationship:

(1) $$i={\mathrm{av}}^{b}$$

where *b* = 1 represents a capacitive controlled mechanism, while *b* = 0.5 corresponds to a diffusion control process. The calculated *b* values for peak 1, 2, and 3 of P‐PbTe/MXene are fitted as 0.85, 0.75, and 0.76 (Figure S8, Supporting Information), respectively, much higher than those of PbTe/MXene (0.71, 0.59, and 0.57) and PbTe (0.66, 0.54, and 0.51), suggesting its capacitive‐controlled K storage behavior.^[^ ^33^ ^]^

The capacitance contribution can be determined by using the following equation:

(2) $$i=k1v+{k2v}^{1/2}$$

where *k*_1_ *v* and *k*_2_ *v* ^1/2^ signify capacitive‐ and diffusion‐controlled contributions, respectively. The capacitance contribution of the P‐PbTe/MXene becomes higher as the scan rate increases (Figure 2g; Figure S9, Supporting Information) and finally reaches the value of 87.0% at 1.1 mV s^−1^ (Figure S10, Supporting Information), significantly larger than those of PbTe/MXene (77.5%) and PbTe (67.7%). Such facile capacitive K storage contributes to the outstanding rate capability and cyclic stability.^[^ ^34^ ^]^

The galvanostatic intermittent titration technique (GITT) was employed to examine the K^+^ ion diffusion kinetics. In comparison to the PbTe/MXene and PbTe electrodes, the P‐PbTe/MXene shows the largest diffusion coefficient during battery operation (Figure 2h; Figure S11, Supporting Information).

The volumetric expansion effect of the materials was further investigated after cycling. As shown in Figure S12a (Supporting Information), the thickness change of the P‐PbTe/MXene electrode ranges from 12 to 15 µm with merely a small swelling rate of 25% after 200 cycles. In contrast, the PbTe shows a huge swelling rate of 171% (Figure S12b, Supporting Information) and obvious irregular cracks (Figure S13, Supporting Information) after 200 cycles, inducing rapid capacity decay and decreased electrical contact.

**Figure 3:**
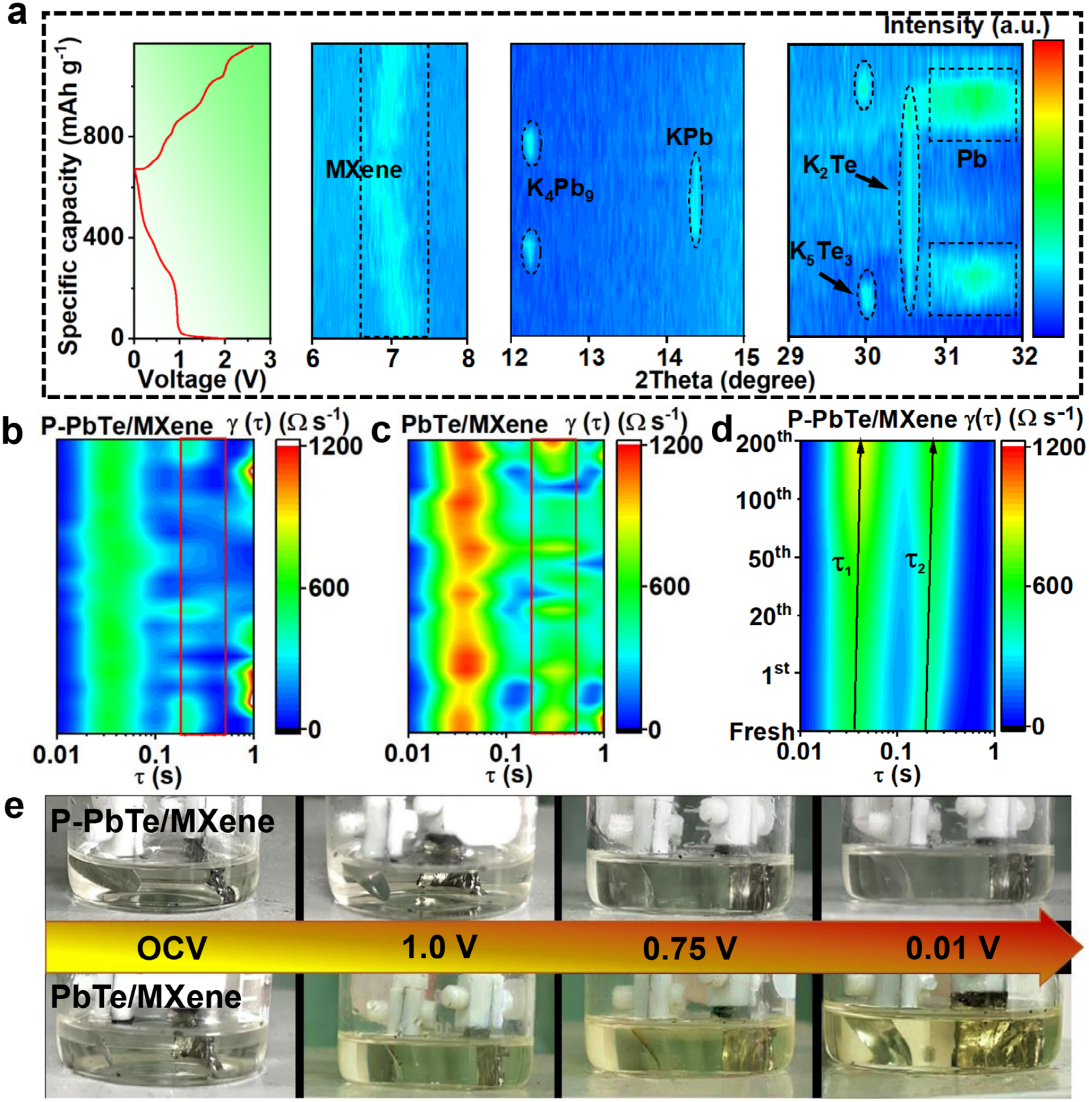
Intensity color map of in situ a) XRD spectra and b‐d) DRT curves during the potassiation/depotassiation processes. e) Optical images of glass batteries during initial discharge.

To clarify the potassium storage mechanism of the P‐PbTe/MXene electrode, in situ XRD and ex situ HRTEM at different discharge/charge states were performed. After fully discharging to 0.01 V, the characteristic (002) peak of MXene gradually shifts from 7.21° to 6.72°, and the corresponding interlayer spacing increases from 1.246 to 1.314 nm (**Figure** **3**a; Figure S14, Supporting Information). Upon charging to 2.6 V, the (002) peak reverts to its initial position, demonstrating its strong self‐buffering capability and high reversibility. The results are consistent with the reversible evolution of interlayer distance measured by HRTEM (Figure S15, Supporting Information).

Upon discharging the P – PbTe/MXene electrode to 1.1 V, an expansion of 0.4% in the lattice spacing of the (200) plane of PbTe relative to its initial state is observed (Figure S16, Supporting Information). This structural change can be mechanistically ascribed to the intercalation of K^+^ ions, leading to the formation of K_x_PbTe.^[^ ^35^ ^]^ As the electrode is further discharged to 0.9 V (Figure 3a; Figure S14, Supporting Information), the new peak at 29.9° can be assigned to the (411) plane of K_5_Te_3_ (PDF#97‐009‐6743), while other peak at 31.2° corresponds to the Pb phase (PDF#97‐005‐2253),^[^ ^36^ ^]^ indicating a conversion reaction to form Pb and K_5_Te_3_. Upon discharge to 0.8 V, the new diffraction peaks at 19.1° and 31.0° can be assigned to the (111) and (220) planes of K_2_Te (PDF#97‐006‐0441), respectively,^[^ ^37^ ^]^ suggesting further conversion of K_5_Te_3_ to K_2_Te. Upon further discharge to 0.4 V, the diffraction peak at 12.2° corresponds to the (‐111) plane of K_4_Pb_9_ (PDF#97‐024‐0029), indicating an alloying reaction from Pb to K_4_Pb_9_. At full discharge condition of 0.01 V, the new peak at 14.3° can be assigned to the (112) plane of KPb (PDF#97‐040‐1436), indicating K_4_Pb_9_ further alloying transforms to the final reduction product of KPb. Upon recharging the electrode to 0.65 V, the KPb is transformed to K_4_Pb_9_. After recharging to 0.86 V, the K_4_Pb_9_ peaks disappear and the Pb peaks reappear, indicating the occurrence of the dealloying reaction of K‐Pb. As it is further charged to 1.5 V, K_2_Te was oxidized to K_5_Te_3_. Notably, the peaks corresponding to PbTe recover after fully recharging to 2.6 V, indicating excellent electrochemical reversibility. Thus, the reversible reaction mechanism of P‐PbTe/MXene with potassium involves the following reversible processes:

(3) $$\mathrm{PbTe}+{\mathrm{xK}}^{+}+{\mathrm{xe}}^{-}\to \mathrm{KxPbTe}$$

(4) $$3\mathrm{KxPbTe}+\left(5-3x\right)K^{+}+\left(5-3x\right)e^{-}\to K5\mathrm{Te}3+3\;\mathrm{Pb}$$

(5) $$K5\mathrm{Te}3+K^{+}+e^{-}\to 3K2\mathrm{Te}$$

(6) $$9\;\mathrm{Pb}+{4K}^{+}+4e^{-}\to K4\mathrm{Pb}9$$

(7) $$K4\mathrm{Pb}9+{5K}^{+}+5e^{-}\to 9\mathrm{KPb}$$

The peak intensity and position of the observed distribution of relaxation times (DRT) profiles derived from the corresponding impedance data (Figure S17a, b, Supporting Information) during the charge/discharge process were used to probe the reaction kinetics and structure stability of the different electrodes. The peaks in the relaxation time range of 10^−2^–0.1 s correspond to the ion transport across the SEI layer, while the peaks in the range of 0.1–1 s represent the charge transfer process and the peaks above 1 s are indicative of the mass transport within the electrolyte phase (Figure S17c,d, Supporting Information).^[^ ^38^ ^]^ Apparently, the time constant of the charge transfer process for P‐PbTe/MXene ranging from 0.15 to 0.23 s (Figure 3b) is much lower than that of PbTe/MXene (0.27–0.45 s, Figure 3c), manifesting the key role of P‐MXene in booting the reaction kinetics. Additionally, the P‐PbTe/MXene demonstrates a less distinct variation in the intensity color map of the DRT curves (Figure 3d; Figure S18 and S19, Supporting Information), highlighting its remarkable reversibility and stability.

To investigate the shuttle effect of polytellurides, the electrolyte after first discharge (Figure 3e) and the separator after long‐term cycling (Figure S20, Supporting Information) of P‐PbTe/MXene system still retain their original appearance, while the PbTe/MXene and PbTe (Figure S21, Supporting Information) show a color change from completely colorless to yellow. The UV–vis spectra of the electrolyte for three different electrodes after discharge to 0.01 V are depicted in Figure S22 (Supporting Information). Compared with the PbTe/MXene and PbTe, the characteristic UV–vis absorbance below 450 nm for polytellurides intermediates is not observed in P‐PbTe/MXene.^[^ ^39^ ^]^ The above results demonstrate that P‐PbTe/MXene can effectively suppress the shuttle and dissolution of polytellurides effectively prevented the dissolution of the polytellurides owing to the strong trapping ability of P‐MXene.

**Figure 4:**
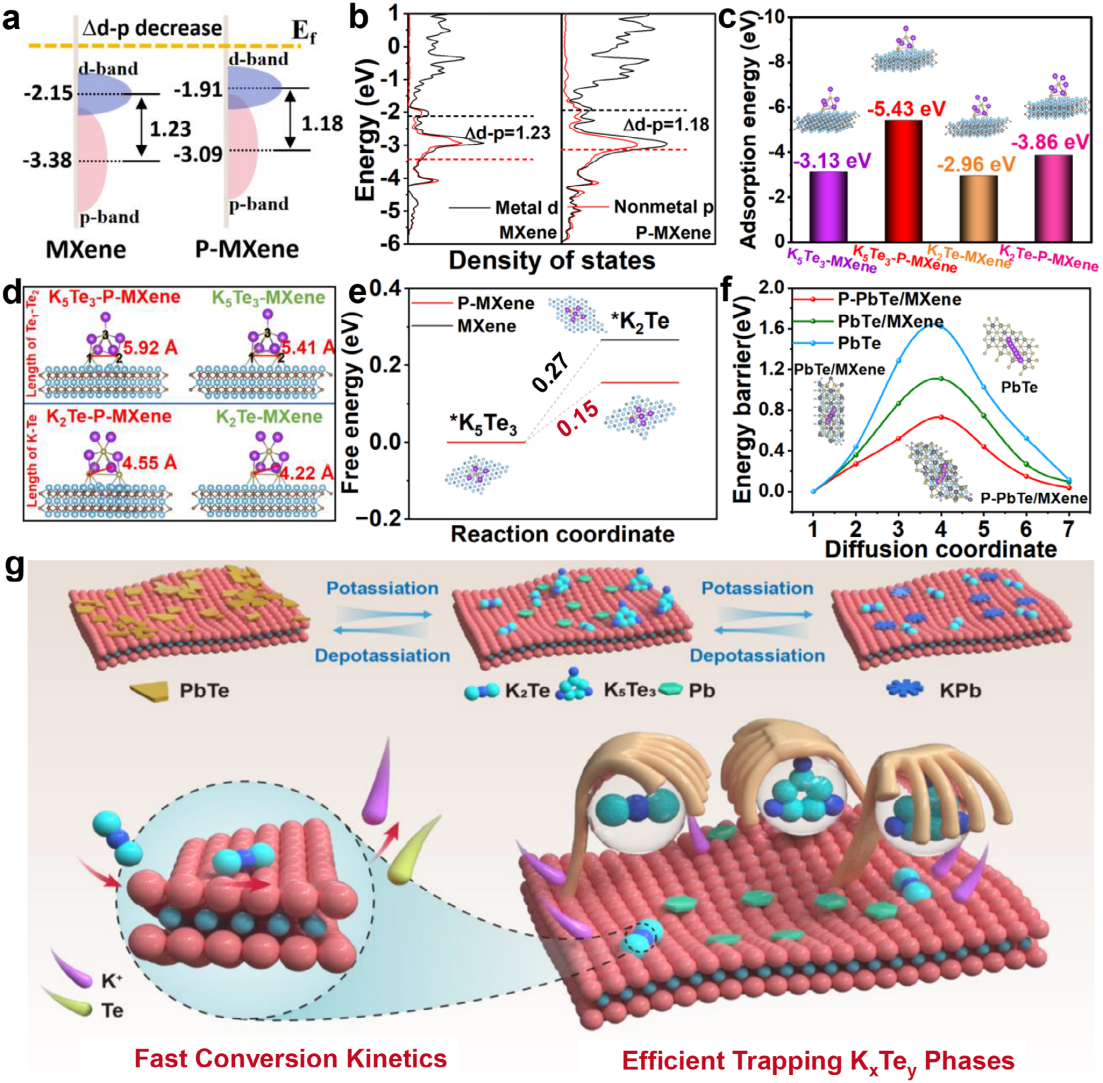
a) Scheme illustration of and b) density of states analysis of metal d and nonmetal p in P‐MXene and MXene. c) DFT calculation results of K_5_Te_3_ and K_2_Te adsorption energy of P‐MXene and MXene. d) Comparison of Te‐Te bond length of K_5_Te_3_ and K‐Te bond length of K_2_Te adsorbed on the surface of P‐MXene and MXene. e) Calculated free energy diagram for K_5_Te_3_ reduction. f) Energy profiles for diffusion processes. g) Scheme illustration of the enhanced “trapping−conversion” function enables ultrastable potassium ion storage for P‐PbTe/MXene.

Density functional theory (DFT) calculations were used to further elucidate the origin of enhanced potassium storage kinetics in P‐PbTe/MXene. The atomic model of the samples is shown in Figure S23 (Supporting Information). In comparison with PbTe/MXene and PbTe, the obtained total density of states (TDOS) of P‐PbTe/MXene reveals the continuous electronic state around the Fermi level and a strong increase in intensity (Figure S24, Supporting Information), implying its typical metallic characteristics and remarkable electronic conductivity. According to the conventional d‐band center model, there are fewer electrons filled in the antibonding orbitals while the d‐band center shifts close to the Fermi level, increasing the bond strength between P‐MXene and the polytellurides intermediates.^[^ ^40^ ^]^ On the other hand, a higher nonmetal p‐band center enhances the participation of p‐band electrons in the valence band, resulting in greater orbital overlap and hybridization with absorbed polytellurides. The calculated d‐band centers (−1.91 eV) of the metal atoms and the p‐band (−3.09 eV) centers of the non‐metals for P‐MXene are larger than those of MXene (−2.15 and −3.38 eV), yielding a lower energy gap (∆d–p) between the p‐band center and d‐band center of electrons (1.18 eV for P‐MXene versus 1.23 eV for MXene, **Figure** **4**a,b). A smaller energy gap between the d‐band and p‐band means a superior electron transfer capability, which accelerates Te_n_^2−^ redox kinetics.^[^ ^41^ ^]^

This viewpoint can be strongly supported by the calculated adsorption energy results. The distances of Ti‐Te bonds in P‐MXene‐K_5_Te_3_ (2.92 Å, Figure S25, Supporting Information) and P‐MXene‐K_2_Te systems (2.93 Å, Figure S26, Supporting Information) are much shorter than those of MXene‐K_5_Te_3_ (3.00 Å) and MXene‐K_2_Te (3.03 Å), suggesting that P‐MXene has stronger adsorption of K_5_Te_3_ and K_2_Te. The adsorption energies of both K_5_Te_3_ (−5.43 eV) and K_2_Te (−3.86 eV) on P‐MXene are much lower than those of MXene (−3.13 and −2.96 eV, Figure 4c). Additionally, the bond lengths of Te‐Te (4.55 Å) of K_2_Te and Te‐Te (5.92 Å) of K_5_Te_3_ on the surface of P‐MXene are longer than those on the MXene (4.22 and 5.41 Å), representing the rapid conversion reaction of polytellurides on P‐MXene (Figure 4d). For the reduction of K_5_Te_3_ to K_2_Te, P‐MXene exhibits much lower reaction‐free energies (0.15 eV) than that of MXene (0.27 eV, Figure 4e), verifying its enhanced K_5_Te_3_ transformation kinetics. The K^+^ ion migration path of the P‐PbTe/MXene and PbTe/MXene interfaces is depicted (Figure S27, Supporting Information. The energy barrier of P‐PbTe/MXene (0.73 eV) is much lower than those of PbTe/MXene (1.11 eV) and PbTe (1.62 eV, Figure 4f), indicating its fast K^+^ ion diffusion kinetics. Based on the above analyses, the fast and stable K^+^ ion storage for P‐PbTe/MXene is illustrated in Figure 4g, which depicts that the shuttle of polytellurides can be suppressed due to enhanced adsorption and catalytic abilities of P‐MXene.

**Figure 5:**
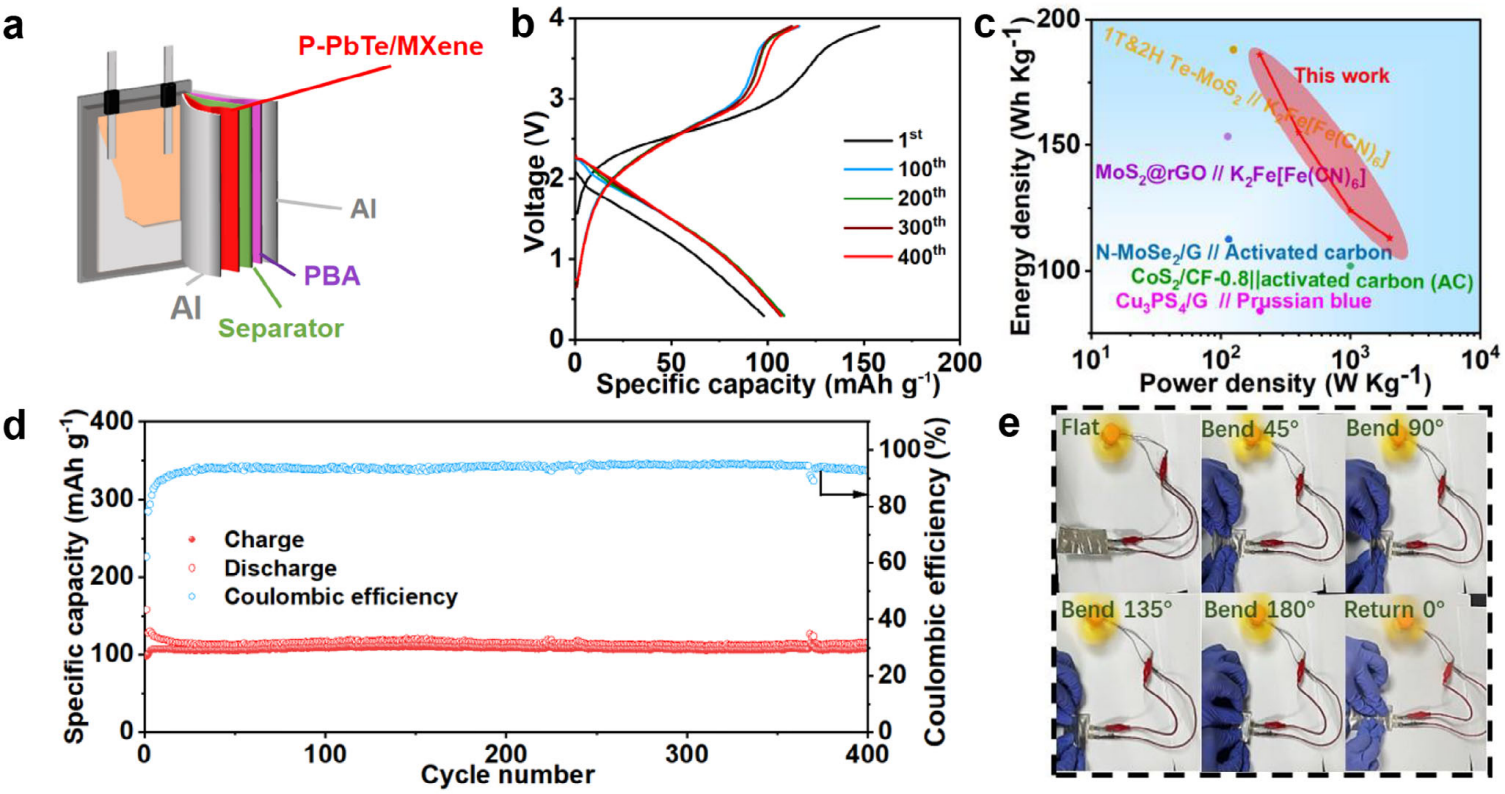
a) Schematic illustration of internal structure for the flexible full cell. b) charge‐discharge profiles of flexible full KIB at 0.1 A g^−1^. c) Ragone plots in comparison with other reports. d) Cycling performance at 0.1 A g^−1^. e) Digital photos showing the flexibility of the full cell under different bending angles.

The excellent potassium storage capacity of P‐PbTe/MXene encourages us to further explore its practical feasibility in flexible full cells. The combination of pre‐intercalated potassium‐treated Prussian Blue Potassium (KPB) cathode and P‐PbTe/MXene anode is illustrated in **Figure** **5**a. The cell was cycled in a voltage range of 0.30–3.90 V based on the discharge/charge profiles of P‐PbTe/MXene and KPB (Figure S28, Supporting Information). The assembled full cell delivers an initial reversible capacity of 119.2 mAh g^−1^ at 0.1 A g^−1^ (Figure 5b). When the current density is increased in steps from 0.1 to 0.2, 0.5, and 1 A g^−1^, the cell still retained reversible capacities of 109.2, 92.7, 81.1, and 72.7 mAh g^−1^, respectively (Figure S29, Supporting Information). The maximum energy and power densities of the full cell are calculated to be 186.0 W h kg^−1^ at 0.1 A g^−1^ and 113.2 W kg^−1^ at 1 A g^−1^, respectively. These values are superior to those of other reported full cells, as shown in Figure 5c and Table S2 (Supporting Information). It is worth noting that after 400 cycles (Figure 5d), the full cell can still provide a reversible capacity of 107.9 mA h g^−1^ with a high coulomb efficiency of nearly 100% at a current density of 0.1 A g^−1^, achieving a reliable electricity supply for a mini electric fan under repetitive folding states (Figure 5e).

## 3 Conclusion

In summary, we have successfully designed a high‐performance P‐PbTe/MXene superstructure as an anode for KIBs using a doping engineering strategy. When P‐doping into the P‐PbTe/MXene architecture, it introduces abundant Te vacancy defects into PbTe and enhances the adsorption and catalytic properties of the MXene substrate. Simultaneously, the excellent capacitive characteristics of P‐PbTe/MXene and the enhanced polytellurides adsorption and conversion capacity, confer high‐rate capability and prolonged cycle life to KIBs. Theoretical simulation calculations further validate the improved electrochemical kinetics of the P‐PbTe/MXene superstructure, as evidenced by its high electronic conductivity, enhanced polytellurides adsorption and catalytic capabilities, and a significant reduction in diffusion barriers for K^+^. As a result, the P‐PbTe/MXene electrode shows a high reversible capacity of 289.1 mAh g^−1^ after 200 cycles at 0.2 A g^−1^, superior rate capability (151.3 mAh g^−1^ at 20 A g^−1^), and superior long‐term cycling stability (180.1 mAh g^−1^ after 2000 cycles at 2.0 A g^−1^). Notably, the P‐PbTe/MXene//KPB flexible full‐cell exhibits high energy density (186.0 Wh kg^−1^ at 0.1 A g^−1^), excellent cycling stability (107.9 mAh g^−1^ at 0.1 A g^−1^ after 400 cycles), and providing power to the device even under different bending degrees. In addition, ex situ characterizations further verify its electrochemical potassium storage mechanism and strong adsorption and catalytic capabilities toward polytellurides. Both hydrothermal synthesis and annealing processes used in our work hold significant promise for large‐scale electrode material fabrication. This work proposes a method to construct ultra‐stable anode materials for KIBs, which may also apply to other MC anode materials.

## Conflict of Interest

The authors declare no conflict of interest.

## Supporting information



Supporting Information

## Data Availability

The data that support the findings of this study are available from the corresponding author upon reasonable request.
