# Supplementary material for: An Enhanced “Trapping−Conversion” Function Enables Ultrastable Potassium Ion Storage
Source: Adv Sci (Weinh). 2025 May 8;12(28):2503332. doi: 10.1002/advs.202503332 (PMC12302578; doi:10.1002/advs.202503332)
Supplement: Supplementary file 1 — Supporting Information [file ADVS-12-2503332-s001.docx]

*Supporting Information*

**An Enhanced “****Trapping−Conversion” Function** **Enables Ultrastable Potassium Ion Storage**

*Zhongquan Wang, Bangjun Wu, Zhenping Qiu, Qingguang Zeng, Aruuhan Bayaguud*, Huirong Wang,*** *Zheng Liu,** *Yiju Li,* Yelong Zhang**

Z. Wang, Prof. H. Wang, Prof. Y. Zhang

School of Renewable Energy, Inner Mongolia University of Technology, Ordos 017010, China

E-mail: wanghr@imut.edu.cn, zhangyelong2008@126.com

Prof. Y. Zhang, Prof. A. Bayaguud

Inner Mongolia Key Laboratory of New Energy and Energy Storage Technology, Hohhot, 010051, China

Prof. A. Bayaguud

School of Materials Science and Engineering, Inner Mongolia University of Technology, Hohhot, 010051, China

Z. Wang, B. Wu, Pro. Z. Qiu, Prof. Q. Zeng, Prof. Z. Liu

School of Applied Physics and Materials, Wuyi University, Jiangmen, Guangdong, 529020, China

Institute of Carbon Peaking and Carbon Neutralization, Wuyi University, Jiangmen, Guangdong, 529020, China

E-mail: zhengliucn@sina.com

Pro. Y. Li

Department of Mechanical and Energy Engineering, Southern University of Science and Technology, Shenzhen, 518055, China

E-mail: liyj6@sustech.edu.cn

**Experimental Methods**

**Preparation of Ti_3_C_2_T_x_ MXene.** Ti_3_C_2_Tx MXene was produced using the *in-situ* HF etching method. Specifically, 1 g of Ti_3_AlC_2_ MAX was slowly added into 30 mL of HF (Aladdin) solution with magnetic stirring for 24 hours at room temperature. After the reaction, the black powder was collected by centrifugation and washed several times with deionized water and ethanol. The powder was dried under vacuum at 60 °C for 12 hours.

**Preparation of** **PbTe/MXene and PbTe.** Under a continuous Ar flow, 3.0 mmol of lead acetate ((CH_3_COO)_2_Pb), 3.0 mmol of sodium tellurite (Na_2_TeO_3_), and 10 mmol hydroxylamine hydrochloride (NH_2_OH·HCl) were dissolved in 30 mL of deionized water. To this solution, 12 mmol of sodium borohydride (NaBH_4_) and 5 mmol of MXene were gradually added under vigorous stirring. The mixture was stirred for 2 hours and then transferred into a 50 mL Teflon-lined autoclave and heated at 200 °C for 24 hours. The cooled product was centrifuged and washed sequentially with deionized water and ethanol to remove ionic residues. The final PbTe/MXene composite was vacuum-dried at 60 °C for 10 hours.

Similarly, the pure PbTe was synthesized without adding MXene.

**Preparation of P-PbTe/MXene.** 0.1 g of resulting PbTe/MXene and 2 g of NaH_2_PO_2_ were placed in two quartz boats, and the quartz boat containing NaH_2_PO_2_ was located on the upstream side of the furnace and annealed at 450 °C for 1 h in an argon atmosphere to obtain P-PbTe/MXene.

**Preparation of potassium Prussian blue (KPB).** In the typical procedure, K_4_Fe(CN)_6_ (1 mmol) was added to 160 mL of deionized water and stirred for 2 hours to prepare solution A. To form solution B, deionized water (40 mL) was supplemented with FeCl_3_ (2 mmol). Next, solution B was slowly dissolved in solution A under stirring for 3 hours, and the reaction mixture was further aged for 24 hours. The obtained precipitates were separated by centrifugation, washed with DI water and ethanol, and then dried in a vacuum oven at 60 °C for 12 hours.

**Material characterizations.** The crystalline structures were determined using an X-ray diffractometer (XRD, Bruker D8) with Cu *Kα* radiation (*λ* = 1.541 Å). The morphology and microstructure were examined using scanning electron microscopy (SEM, JEOL, JSM-5612LV) and high-resolution transmission electron microscopy (HRTEM, JEOL JEM-2100F). The chemical state of the elements was determined using X-ray photoelectron spectroscopy (XPS, Kratos XSAM-800) with a monochromatic Al *Kα*_1_ X-ray. The BET results were obtained by conducting nitrogen adsorption-desorption measurements at 77 K using ASAP 2010 and were calculated according to the Brunauer-Emmett-Teller (BET) theory. The presence of vacancies was investigated using electron paramagnetic resonance (EPR) spectroscopy with a Bruker EMXplus-10/12 instrument. Cyclic voltammetry (0.1-1.1 mV s^−1^) and electrochemical impedance spectroscopy (100 kHz-0.1 Hz) were performed on a VMP3 workstation. Galvanostatic charge/discharge (0.01-2.6 V) and GITT protocols (0.2 A g^–1^ pulse/420 s, 1200 s relaxation) were executed using a LAND CT2001A system.

**Half- and full-cell construction.** For half-cell preparation, the working electrode slurry was formulated by homogenizing active material, polyvinylidene fluoride (PVDF), and Super P carbon additive (80:10:10 wt%) in N-methyl-2-pyrrolidone (NMP) under 10 hours magnetic stirring. The resulting mixture was blade-coated onto Cu foil (1.0 mg cm^−2^ active mass loading) and vacuum-dried at 60 °C for 8 hours. CR2032 coin cells were assembled in an argon-protected glovebox, utilizing potassium foil counter electrodes and glass fiber separators (Whatman GF/F). The electrolyte comprised 0.8 M KPF_6_ dissolved in ethylene carbonate (EC)/ diethyl carbonate (DEC) (1:1 v/v).

Flexible full cells integrated KPB cathodes (80:10:10 KPB/Super P/PVDF on Cu foil) with pre-conditioned P-PbTe/MXene anodes. Both electrodes underwent five activation cycles (0.1 A g^−1^) in half-cell configurations: cathodes between 2.0-4.0 V and anodes at 0.01-2.6 V to establish stable interfaces. The cathode-limited design maintained a 3:1 mass ratio (KPB:anode ) with voltage cutoffs 0.3-3.9 V. The energy/power densities calculated via: E = ∫ V dQ and P = E / t, where E, V, Q, P, and t denote energy density, operating voltage, capacity, power density, and discharge duration, respectively.

**DFT calculations.** Calculations based on first-principles density functional theory (DFT) were executed utilizing the Vienna Ab initio Simulation Package (VASP) in conjunction with the Projector Augmented Wave (PAW) methodology. The exchange-correlation functional was managed within the parameters of the Generalized Gradient Approximation (GGA), adopting the Perdew-Burke-Ernzerhof (PBE) functional. We implemented a plane wave basis set with an energy cutoff set at 500 eV, and the geometric relaxation was carried through until the forces acting on each atom were less than 0.03 eV/Å. The sampling of the Brillouin zone was conducted using a 1 × 1 × 1 k-point grid for all modles.To assure rigorous consistency, calculations were performed until the energy convergence threshold was less than 10-5 eV. To effectively isolate periodic structures and preclude their interaction, a vacuum buffer of 15 Å was inserted along the z-axis. The unit cell of PbTe and PbTe/MXene is defined by the lattice constants: *a* = 9.17530 Å, *b* = 9.17530 Å, *c* = 27.93310 Å, *α* = *β* = 90°, and *γ* = 120°; The unit cell of K_2_Te-MXene, K_2_Te-P-MXene, K_5_Te_3_-MXene, K_5_Te_3_-P-MXene, Te-MXene, and Te-P-MXene is defined by the lattice constants: *a* = 15.35320 Å, *b* = 15.35320 Å, *c* = 29.00000 Å, *α* = *β* = 90°, and *γ*= 120°.The unit cell of PbTe-K-MXene, P-PbTe-K-MXene (V_Te_), and K-PbTe is defined by the lattice constants: *a* = 9.17530 Å, *b* = 18.35060 Å, *c* = 27.93310 Å, *α* = *β* = 90°, and *γ* = 120°.

**The calculation process of the diffusion coefficient (D_K+_).** The diffusion coefficients were estimated from the GITT potential profiles using Fick's second law, which is described by the following equation:

*D = 4/πτ ((m_a_ V_M_)/(M_a_ S))^2^ (ΔE_s_/ΔE_τ_)^2^* (1)

In the equation, *τ* represents the duration of the current pulse, *m_a_* and *M_a_* denote the active mass and molar mass of the active material in the electrode, and *V_M_* represents the molar volume. The value of *M_a_/V_M_* can be obtained from the density of the electrode materials. Furthermore, the values of *τ*, *ΔE_s_*, and *ΔE_τ_* can be obtained from the GITT curves.

**Figure S1.** SEM images of a) Ti_3_AlC_2_ MAX and b) Ti_3_C_2_T_x_ MXene. c)XRD patterns of Ti_3_AlC_2_ MAX and Ti_3_C_2_T_x_ MXene.

**Figure S2.** SEM image of PbTe/MXene.

**Figure S3.** a) TEM, b) elemental mapping images, and c) EDS of P-PbTe/MXene.

**Figure S4.** a) XPS survey spectra of P-PbTe/MXene. b) High-resolution Te 3d XPS spectra of P-PbTe/MXene and PbTe/MXene.

**Figure S5.** a) Comparison of EPR of P-PbTe/MXene, PbTe/MXene, and PbTe. b) N_2_ adsorption-desorption isotherms of P-PbTe/MXene, PbTe/MXene, PbTe, and MXene.


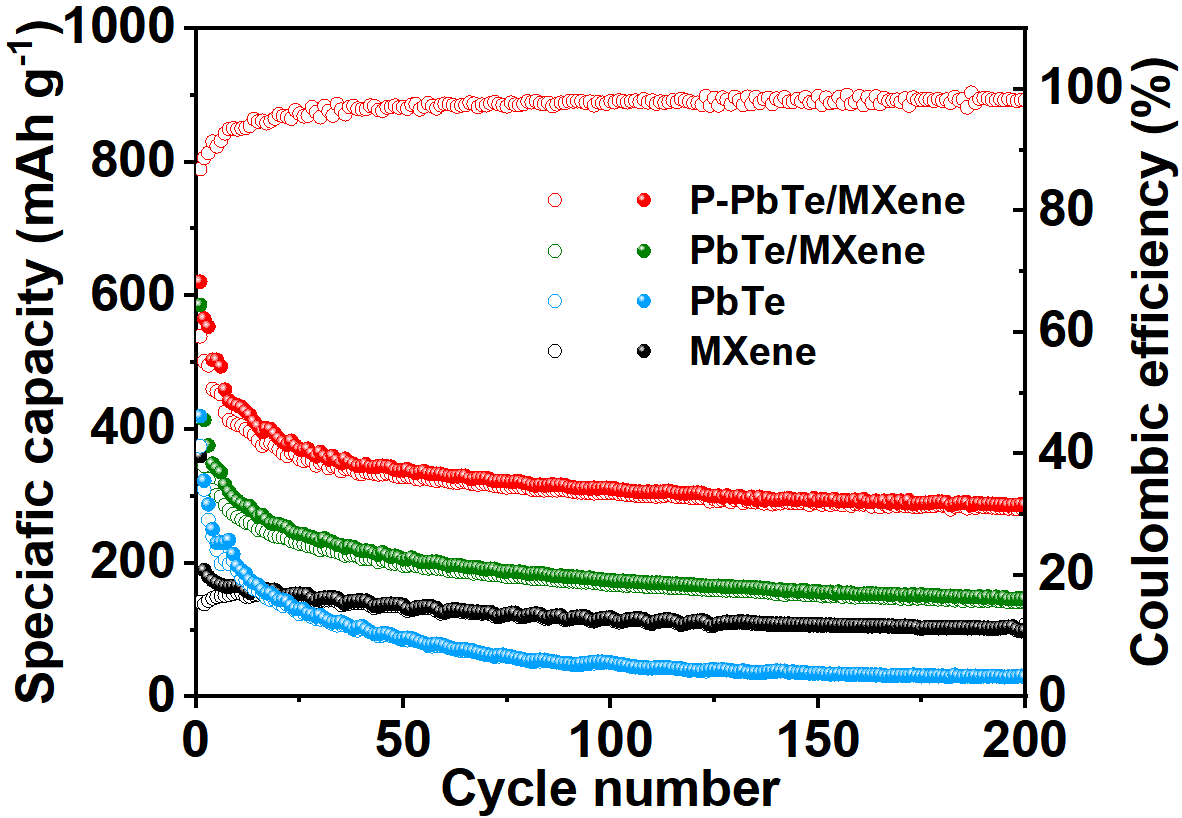


**Figure S6.** Cycling performance of P-PbTe/MXene at 0.2 A g^−1^.

**Figure S7.** CV curves of a) P-PbTe/MXene, b) PbTe/MXene, and c) PbTe at different scan rates.

**Figure S8.** Log (*i*) vs. Log (*v*) plots of redox peaks for P-PbTe/MXene, PbTe/MXene, and PbTe.

**Figure S9.** Contribution ratio of capacitive behaviors at different scan rates for a) PbTe/MXene and b) PbTe.

**Figure S10.** The capacitive (shadow) contribution at 1.1 mV s^−1^ for a) P-PbTe/MXene, b) PbTe/MXene, and c) PbTe.

**Figure S11.** GITT curves for a) P-PbTe/MXene, b) PbTe/MXene, and c) PbTe.


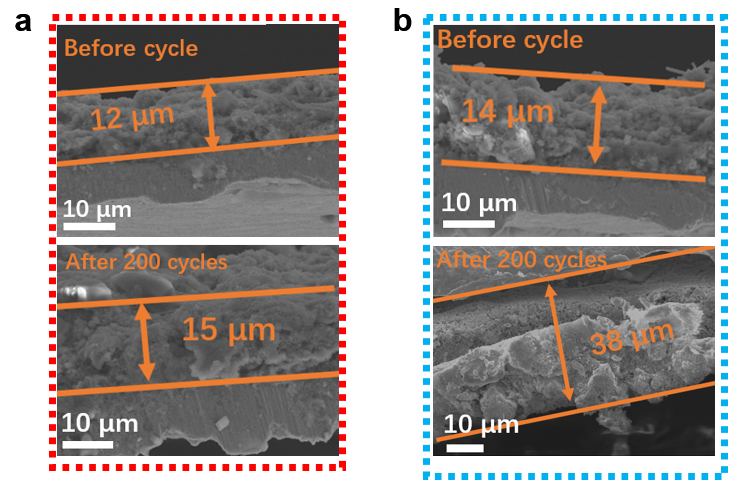


**Figure S12.** SEM images of pristine and cycled a) P-PbTe/MXene and b) PbTe electrodes.


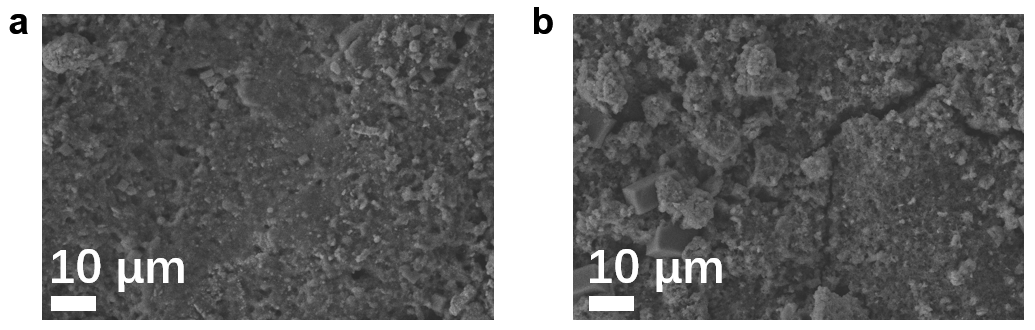


**Figure S13.** SEM images of a) P-PbTe/MXene and b) PbTe/MXene after 200 cycles at 0.2 A g^−1^.

**Figure S14.** *In situ* XRD spectra of the P-PbTe/MXene electrode during the potassiation/ depotassiation processes.


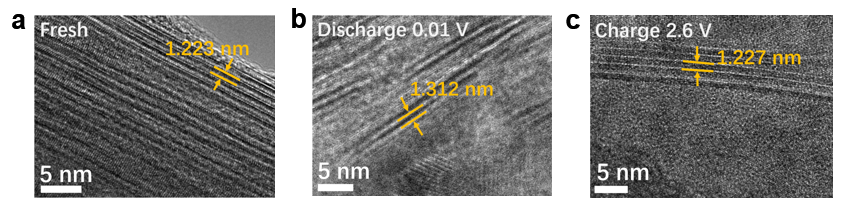


**Figure S15.** a-c) The interlayer spacing of MXene at various discharge/charge depths.


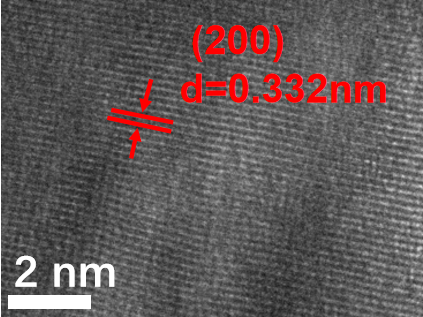


**Figure S16.** HRTEM image of P-PbTe/MXene at a discharge depth of 1.1V.

**Figure S17.** *In-situ* EIS plots and corresponding DRT curves of a, c) P-PbTe/MXene and b, d) PbTe/MXene during the potassiation/depotassiation processes.

**Figure S18.** EIS plots of a) P-PbTe/MXene and b) PbTe/MXene at different cycles.


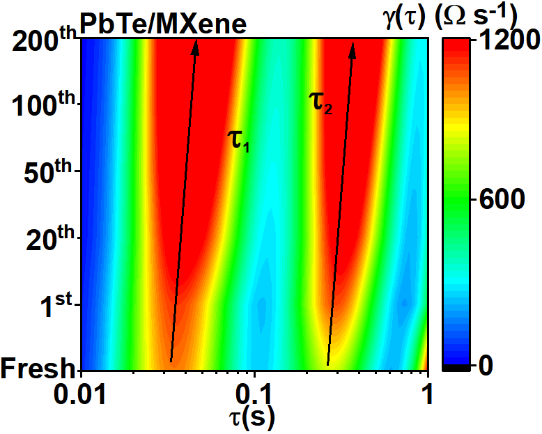


**Figure S19.** Intensity color map of the DRT curves for PbTe/MXene at different cycles.


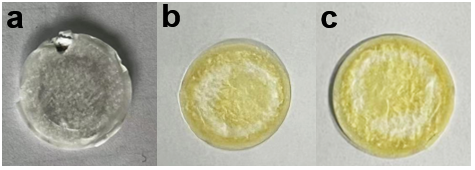


**Figure S20.** Color comparison of separators after 200 cycles for a) P-PbTe/MXene, b) PbTe/MXene, and c) PbTe.

**Figure S21.** Optical images of glass battery during initial discharge of PbTe.

**Figure S22.** UV-vis absorption spectra of electrolytes in different battery after initial discharge.


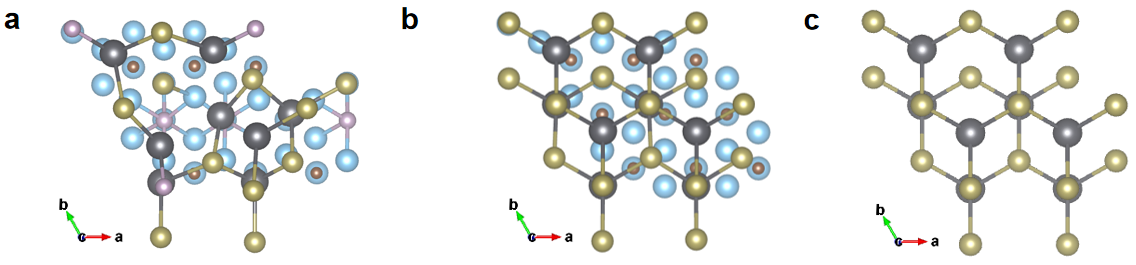


**Figure S23.** Atomic model diagram of a) P-PbTe/MXene, b) PbTe/MXene, and c) PbTe.

**Figure S24.** Density of states of P-PbTe/MXene, PbTe/MXene, and PbTe.

**Figure S25.** Comparison of Te-Ti bond length of K_5_Te_3_ clusters adsorbed on the surface of a) P-MXene and b) MXene.

**Figure S26.** Comparison of Te-Ti bond length of K_2_Te clusters adsorbed on the surface of a) P-MXene and b) MXene.


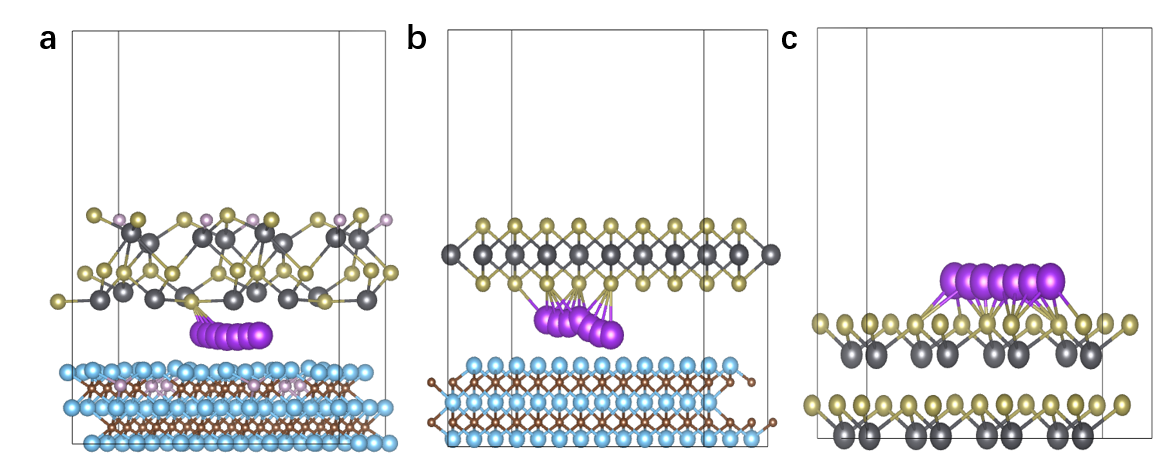


**Figure S27.** The pathway for K diffusion in a) P-PbTe/MXene, b) PbTe/MXene, and c) PbTe.

**Figure S28.** The discharge/charge profiles of the P-PbTe/MXene anode and KPB cathode at a current rate of 0.2 A g^-1^.

**Figure S29.** Rate capability of the full cell.

**Table S1.** Comparison of electrochemical performance of various transition metal chalcogenides-based anode materials for PIBs.

| **Active materials** | **Current density (A g^−1^)** | **Cycle number** | **Capacity**  **(mAh g^−1^)** | **Ref.** |
| --- | --- | --- | --- | --- |
| P-PbTe/MXene | 2.0 | 2000 | 180.1 | This work |
| G@Y–S FeS_2_@C | 1.0 | 1000 | 162.0 | [1] |
| SnS_2_@C-2 | 2.0 | 1000 | 183.1 | [2] |
| MoS_2_–C/rGO | 2.0 | 2000 | 161.6 | [3] |
| NGr/2H(1T)-MoS_2_ | 0.5 | 1000 | 151.0 | [4] |
| CoS_2_/CuCo_2_S_4_@NCs | 1.0 | 1000 | 112.0 | [5] |
| CoSe_2_-NPC@CNS) | 1.0 | 1000 | 165.1 | [6] |
| CuSbS_y_@C | 1.0 | 1000 | 174.5 | [7] |
| Bi_2_Se_3_@NC | 1.0 | 2000 | 130.5 | [8] |
| FeS_2_@C | 2.0 | 1000 | 164.0 | [9] |

**Table S2.** Comparison of energy density and power density of various transition metal dichalcogenides-based materials for full PIBs.

| **Electrode materials** | **Energy density and power density** | **Ref.** |
| --- | --- | --- |
| P-PbTe/MXene // KPB | 186 Wh kg^−1^ and 200 W kg^−1^  155 Wh kg^−1^ and 400 W kg^−1^  124 Wh kg^−1^ and 1000 W kg^−1^  113 Wh kg^−1^ and 2000 W kg^−1^ | This work |
| CoS_2_/CF-0.8// Activated carbon (AC) | 102 Wh kg^−1^ and 999 W kg^−1^ | ^[10]^ |
| MoS_2_@rGO // K_2_Fe[Fe(CN)_6_] | 153 Wh kg^−1^ and 112 W kg^−1^ | ^[11]^ |
| Cu_3_PS_4_/G // Prussian blue | 84 Wh kg^−1^ and 205 W kg^−1^ | ^[12]^ |
| N-MoSe_2_/G // Activated carbon | 115 Wh kg^−1^ and 112 W kg^−1^ | ^[13]^ |
| 1T&2H Te-MoS_2_ // K_2_Fe[Fe(CN)_6_] | 188 Wh kg^−1^ and 125 W kg^−1^ | ^[14]^ |

**References**

[1] Y. Zhao, J. Zhu, S. J. H. Ong, Q. Yao, X. Shi, K. Hou, Z. J. Xu, L. Guan, *Adv. Energy Mater.* **2018**, 8, 1802565.

[2] D. Li, L. Dai, X. Ren, F. Ji, Q. Sun, Y. Zhang, L. Ci, *Energ Environ Sci.* **2021**, 14, 424.

[3] J. Li, F. Hu, H. Wei, J. Hei, Y. Yin, G. Liu, N. Wang, H. Wei,*Compos Part B-Eng* **2023**, 250, 110424.

[4] P. Zhang, Y. Yang, X. Duan, S. Zhao, C. Lu, Y. Shen, G. Shao, S. Wang, *Nano Energy* **2020**, 78, 105352.

[5] G. Suo, S. Musab Ahmed, Y. Cheng, J. Zhang, Z. Li, X. Hou, Y. Yang, X. Ye, L. Feng, L. Zhang, Q. Yu, *J. Colloid Interface Sci.* **2022**, 608, 275.

[6] Q. Liu, X. Tan, X. Li, Y. Li, X. Han, S. Cui, D. Xu, Y. Liu, R. Wang, Q. Zhao, M. Wu, *J. Energy Storage* **2024**, 87, 111449.

[7] P. Hu, Y. Dong, G. Yang, X. Chao, S. He, H. Zhao, Q. Fu, Y. Lei,*Batteries* **2023**, 9, 238.

[8] X. Sun, B. Zhang, M. Chen, L. Wang, D. Wang, R. Man, S. Iqbal, F. Tian, Y. Qian, L. Xu, *Nano Today* **2022**, 43, 101408.

[9] Y. Du, W. Weng, Z. Zhang, Y. He, J. Xu, J. Sun, J. Liao, J. Bao, X. Zhou, *ACS Materials Lett.* **2021**, 3, 356.

[10] P. Wang, T. Hu, Y. Guo, Y. Cui, R. Wang, A. Yang, Y. Huang, X. Wang, *J. Alloy. Compd.* **2024**, 970, 172618.

[11] S. Chong, L. Sun, C. Shu, S. Guo, Y. Liu, W. Wang, H. K. Liu, *Nano Energy* **2019**, 63, 103868.

[12] S. F. Ho, H. Y. Tuan, *Chem. Eng. J.* **2023**, 452, 139199.

[13] Y. Yi, Z. Sun, C. Li, Z. Tian, C. Lu, Y. Shao, J. Li, J. Sun, Z. Liu, *Adv. Funct. Mater.* **2020**, 30, 1903878.

[14] W. Kang, R. Xie, Y. Wang, C. An, C. Li, *Nanoscale* **2020**, 12, 24463.
